# Emergency Psychiatry before and during COVID-19 pandemia

**DOI:** 10.1192/j.eurpsy.2022.709

**Published:** 2022-09-01

**Authors:** A. Giménez-Palomo, M. Sagué, G. Fico, M. Gómez-Ramiro, M. Vázquez

**Affiliations:** 1 Hospital Clínic, Department Of Psychiatry And Psychology, University Of Barcelona, Barcelona, Spain; 2 Complejo Hospitalario Universitario de Pontevedra. SERGAS., Department Of Psychiatry, Pontevedra, Spain

**Keywords:** Emergency Service, substance use disorders, psychiatry, Coronavirus

## Abstract

**Introduction:**

The COVID-19 pandemic had a significant impact worldwide. Consultations in the Emergency Service of the Hospital Clínic of Barcelona varied in terms of reasons for consultations, psychopathology, and other aspects, before and after the pandemic.

**Objectives:**

To examine changes in the profile of patients admitted before and during the COVID-19 pandemic to our Psychiatric Emergency Service.

**Methods:**

All children, adolescent and adult psychiatric inpatients admitted from December 4^th^ 2019 to March 31^st^ 2021 to the Psychiatric Emergency Service of Hospital Clínic of Barcelona, Spain, were retrospectively included for analysis and divided into two groups –groups 1 or 2- including the first one all patients who attended before lockdown and the second group those who attended during the pandemic.

**Results:**

A total of 1991 patients were included -1224 in the first group and 767 in the second group. The majority of patients were male (52.08%), with a mean age of 41.21 years (SD 16.53). A proportion significantly higher of men was found in the second group (p<0.05). The proportion of patients consulting with substance use disorders was significantly higher in the second group (p<0.05). Patients from the second group presented a significantly higher proportion of admissions in an acute psychiatric ward (p<0.05), and also a significantly higher proportion of consultations of patients with dementia (p<0.05).

**Conclusions:**

The COVID-19 pandemic lead to a significant reduction in the overall consultations, with a higher proportion of severe cases. The lack of availability of caregivers and telework might have influenced the increase in consultations of patients with dementia.

**Disclosure:**

AG has received travel and financial support from Janssen, Otsuka-Lundbeck and Angelini, and research support from Instituto de Salud Carlos III, and declares no support related with the subject of this presentation.

